# Simulating photodissociation reactions in bad cavities with the Lindblad equation

**DOI:** 10.1063/5.0033773

**Published:** 2020-12-21

**Authors:** Eric Davidsson, Markus Kowalewski

**Affiliations:** Department of Physics, Stockholm University, Albanova University Center, SE-106 91 Stockholm, Sweden

## Abstract

Optical cavities, e.g., as used in organic polariton experiments, often employ low finesse mirrors or plasmonic structures. The photon lifetime in these setups is comparable to the timescale of the nuclear dynamics governing the photochemistry. This highlights the need for including the effect of dissipation in the molecular simulations. In this study, we perform wave packet dynamics with the Lindblad master equation to study the effect of a finite photon lifetime on the dissociation of the MgH^+^ molecule model system. Photon lifetimes of several different orders of magnitude are considered to encompass an ample range of effects inherent to lossy cavities.

## I Introduction

Motivated by recent experimental advancements,^1–3^ state-of- the-art computational tools are presently employed to consider the interaction between quantized electromagnetic radiation and a diverse range of matter systems in models reminiscent of the early Jaynes-Cummings model.^4^ Examples of such matter systems include quantum dots,^5^ excitons,^6^ ensemble systems,^7–9^ Bose–Einstein condensates,^10^ superconductors,^11^ and molecules of different sizes and complexity.^12^

Central to these studies, be it experimental or theoretical, is the macro- or nanoscopic material structure giving rise to electromagnetic field-modes in a confined space, henceforth called a cavity structure, though they are not always reminiscent of the widely used Fabry–Perot cavity. The material and geometry of these cavity structures determine the characteristics of the light–matter interaction, and precise tailoring enables a unique possibility to probe or control phenomena that take place on the scale of single particles. Examples of such phenomena include chemical reactions,^1^ photo-dissociation,^9^ electron transport,^13,14^ isomerization,^15^ and heat-transfer between molecules.^16^

In theoretical works such as this, the properties of the cavity structure are typically translated into parametric values, such as the mode frequency and the vacuum electric field strength,^17,18^ entering the model of the system. The influence of these parameters is well studied and generally well understood.

However, not all parameters describing the cavity structure have gained equal attention. Effects arising from a finite lifetime for field excitations, also known as the cavity Q-factor or photon decay rate, have only recently been the main focus in a few ground-breaking studies,^19–21^ and dissipative effects, in general, have been described as a challenge for computational methods^1^.

Theoretical studies often assume an infinite lifetime,^15^ which can be appropriate but introduces inaccuracies when used to model conditions where finite photon lifetimes are de facto non-negligible. For instance, this is typically the case for plasmonic nano-cavity structures,^11,22^ such as ultra-thin metallic gaps^23^ or nano-gap antennas,^24,25^ where the desire to concentrate a strong electromagnetic field in a small volume inevitably leads to short-lived excitations of the field^1^.

Another previously employed strategy is to incorporate a Hamiltonian coupling between affected states and a reservoir ofsimple systems.^26^ However, this allows the energy to return from the reservoir and affect the evolution of the system, which therefore makes an approximative model for electromagnetic energy being lost into the environment. Additionally, photon decay processes inevitably introduce decoherence into the system, which makes an approach with a pure state wave function insufficient.^27^ Instead, a density matrix formalism is required for the time evolution of these statistically mixed states, and the Schrödinger equation is generalized to some variety of a master equation. Here, we use the Markovian Lindblad master equation, where the decay rate of field excitations will enter through the cavity decay rate *k*,^27,28^

(1) $$\partial_{t}\hat{\rho }=-\frac{\text{i}}{\hbar }[Ĥ,\hat{\rho }]+\sum_{n}\kappa_{n}({\hat{L}}_{n}\hat{\rho }{\hat{L}}_{n}^{†}-\frac{1}{2}{\left[{\hat{L}}_{n}^{†}{\hat{L}}_{n},\hat{\rho }\right]}_{+}).$$

However, using the density matrix, $\hat{\rho }$, increases the computational cost considerably compared to a wave function based approach.

Recent publications^19–21^,^29,30^ on cavity decay do not deal directly with the Lindblad equation formalism; instead, the underlying formalism is a non-Hermitian evolution of a pure state, which is shown to be appropriate for both relaxation dynamics^19^,^21^ and isomerization.^20^ However, for general applications, the method omits some phenomenological processes, such as decoherence and time evolution of statistically mixed states, and the impact of this exclusion depends on the type of system and is observable under investigation.

In this paper, we investigate the molecular dissociation of the MgH^+^ molecule after excitation to an unbound electronic state and in the presence of a lossy cavity structure. Here, the photon decay will populate intermediate states that also contribute to the dissociation. Thus, a wave function based approach is not suitable for this type of problem (see the discussion at the end of Sec. II). Instead, the Lindblad master equation is used to capture the behavior of the system and deliver accurate results. We investigate how the photon lifetime and cavity vacuum field strength affect the photostability of the MgH^+^ molecule, and the photochemical reaction mechanisms of the coupled light–matter system are analyzed.

## II System and Model

The Hamiltonian in Eq. (1) is a molecular Jaynes–Cummings type Hamiltonian^9^ modeling vibrationally and electronically excited states in the presence of a lossy cavity mode. It is composed of *Ĥ_m_* for the molecule, *Ĥ* for the cavity mode, and the light–matter interaction *Ĥ_cm_*,

(2) $$Ĥ={Ĥ}_{m}+{Ĥ}_{c}+{Ĥ}_{cm}\;.$$

**Fig. 1:**
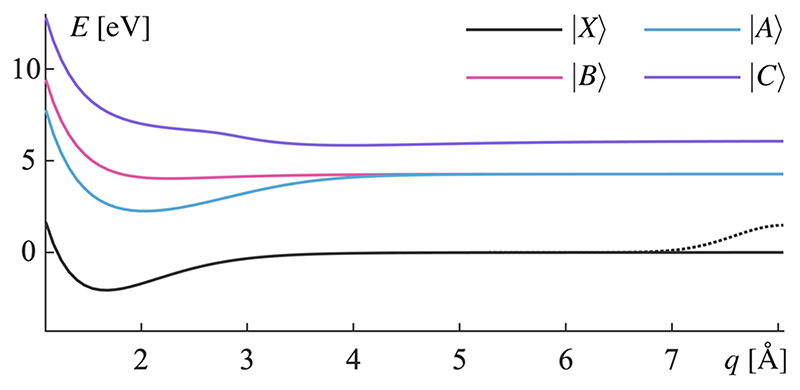
The four lowest electronic states in MgH^+^. Each potential energy surface is implemented with an absorbing potential, whose Gaussian shape and relative size are shown with the dotted line on the black curve.

We assume the cavity Born–Oppenheimer approximation,^31^ the rotating wave approximation, and the dipole approximation for a spatially fixed molecule. Excited electronic and vibrational states of the molecule are described on one-dimensional potential energy surfaces. The four lowest electronically excited states of MgH^+^ are considered (see Fig. 1). Wave-packets approaching the dissociation limit are absorbed by an imaginary potential. The cavity mode is modeled as a single mode with a photon energy of 4.3 eV (285 nm). Comprehensive details about the Hamiltonian are found in Appendix A.

The states of the combined molecule-cavity system are expressed as a product state, |*n,M*〉 = |*n*〉 ⊗ |*M*〉, where |*n*〉 ∈ {|0〉, |1〉, |2〉,…} are the Fock states of the cavity mode and |*M*〉 ∈ {|*X*〉, |*A*〉, |*B*〉, |*C*〉} are the electronic states of MgH^+^. All product states that have distinctly higher energy than the initial state, |0,*C*〉, will never be populated under the rotating wave approximation and are removed from the description. The full Hamiltonian in Eq. (2) is then expressed in the basis of eight states {|0,*X*〉, |1,*X*〉, |0,*A*〉, |0,*B*〉, |2,*X*〉, |1,*A*〉, |1,*B*〉, |0,*C*〉} covering an energy range of ∼10 eV.

**Fig. 2:**
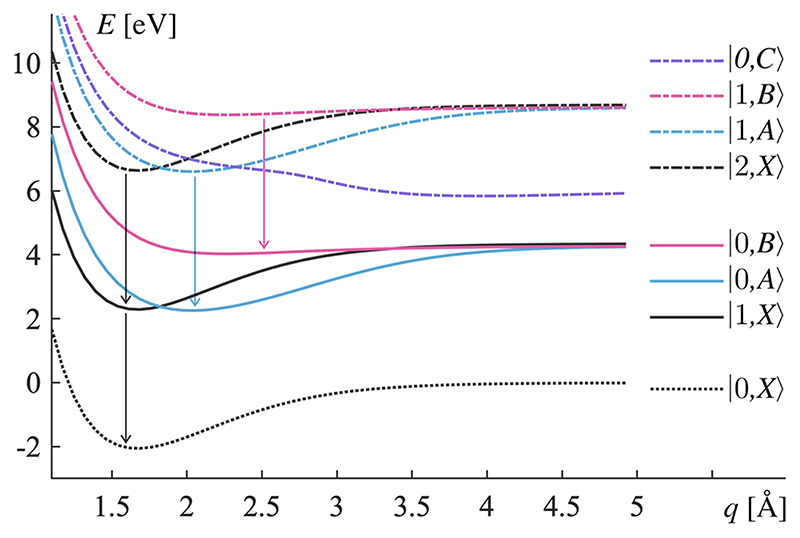
Potential energy surfaces associated with the basis states for electronic and cavity degrees of freedom. The initial state is the pure molecular excitation, |0,*C*〉. Arrows indicate which states are coupled by Lindblad decay operators. States shown as dashed curves are referred to as the double excitation subspace. States shown as solid curves are referred to as the single excitation subspace. The state shown as a dotted curve is referred to as the ground-state subspace. The two lower subspaces (ground state and single excitation) are occasionally considered as a group and referred to as the decohering subspaces.

The product states are grouped into three subspaces: the ground state subspace, the single excitation subspace, and the double excitation subspace. The corresponding potential energy curves are shown in Fig. 2. This partition will later be used for analyzing the dynamics of the system. Under the rotating wave approximation, states from different excitation subspaces are not coupled, which simplifies the Hamiltonian.^32^ Only states within the same excitation subspaces are coupled and exhibit curve crossings, as shown in Fig. 2.

The photon decay, as modeled by the Markovian Lindblad master equation, assumes that the photon is lost irrevocably from the system^28^ (either into free space or by absorption at the surrounding bulk material).

In the product basis, these unidirectional interactions are identified by, wherever possible, decreasing the photon number by one. This yields four transitions: |1,*B*〉 → |0,*B*〉, → |1,*A*〉 → |0,*A*〉 → |2,*X*〉 → |1,*X*〉, and |1,*X*〉 → |0,*X*〉, which are indicated by vertical arrows in Fig. 2. Each of these unidirectional interactions forms a non- Hermitian Lindblad jump operator. The decay rates {*K_n_*} from the general case in Eq. (1) are here the same value, *k*, for all photons in the mode, and *k* is determined for any particular cavity structure. Since the state | 2,X〉 has two photons, the associated annihilation operator will introduce a factor of 2 in the Lindblad operator, effectively increasing the decay rate proportional to the energy increase in the field,

(3) $$\{\begin{array}{l} L_{1}=|0,B\text{〉}\text{〈}1,B|,\\ L_{2}=|0,A\text{〉}\text{〈}1,A|,\\ L_{3}=2|1,X\text{〉}\text{〈}2,X|,\\ L_{4}=|0,X\text{〉}\text{〈}1,X|. \end{array}$$

This model includes no Lindblad de-phasing operators, such as the effects derived from weak interactions between the molecule and its environment, which are assumed to be negligible on the timescale for molecular dissociation of MgH^+^.

The initial state for the simulation, ${\hat{\rho }}_{0}$, is formed from the pure state |0, C,*ψ*(*q*)〉, where the component *ψ*(*q*) is the nuclear vibrational wave function, created by weighting the ground-state wave function of |*X*〉 with the transition dipole moment between ∣*X*⟩↔∣*C*⟩, which corresponds to a vertical excitation,

(4) $${\hat{\rho }}_{0}=|0,C,\psi (q)\text{〉}\text{〈}0,C,\psi (q)|.$$

This method provides a consistent initial condition of a fully excited molecule without the introduction of additional parameters. With *Ĥ*, $\left\{{\hat{L}}_{n}\right\}$, *k*, and ${\hat{\rho }}_{0}$, the model is fully defined, and the time evolution of Eq. (1) can be simulated as described in Sec. III.

As a general remark, to minimize computational cost, the Lindblad master equation can be reduced to a non-Hermitian Schrodinger equation employing an absorbing potential, given the following three conditions: The Hamiltonian does not couple any state in the subspace to other states that are not in the subspace, the initial state projected onto that subspace is a pure state, and the subspace is not the recipient of decaying states. This motivates the method in previous studies,^19–21^ but in this study, these conditions only apply to the doubly excited subspace (see Fig. 2 for definitions of subspaces). The main issue is that a straightforward reduction to a Schrodinger equation only accounts for the removal of population, and not the re-population that happens in the single excitation subspace, and ground-state subspace. The re-population is necessary for this study since a dominant contribution to the observable occurs after it. Additionally, when the decay from the Lindblad operators is transferring population between subspaces, the state is decohering and re-populating the receiving subspaces as a statistical mixture of states. These mixed states display a suppressed interference when evolving on the potential energy surfaces, an effect that is further discussed in relation to our data in Sec. IV. For these reasons, time evolution of a non-Hermitian Schrodinger equation, which only accommodates pure states, is not well suited to our system. Instead, the Lindblad equation will describe these influential effects.

## III Methods

The potential energy curves (see Fig. 1) are calculated with the program package Molpro^33^ at the CASSCF(12/10)/MRCI/ROOS level of theory.^34,35^ The time evolution of the Lindblad equation is done numerically with a Runge–Kutta scheme as it is implemented in the differential equation solver *ode*45^36^ in Octave^37^. The density operator $\hat{\rho }$ is represented on a numerical grid for the nuclear coordinate *q* with 96 grid points for each of the included states | *n*, *M*〉. The density matrix is propagated for 500 fs, a duration selected to reflect the relevant timescale of the MgH^+^ dissociation. Due to the increased computational cost associated with the Lindblad equation, a strategy was developed where Lindblad operators could be summed ahead of time evolution, reducing both memory requirements and computational cost (see the derivation and details about this strategy in Appendix B).

The accuracy of the method is tested against time evolution with our in-house software package QDng using the Chebyshev propagation method,^38^ and good agreement between the two was found (see Appendix C for benchmarking results and further details about the implementation of the numerical method).

## IV Results and Discussion

Initially, the system is vertically excited in full to the state | 0, C〉, which corresponds to a dissociative state of the MgH^+^ molecule. Since this state has no field excitations, the initial state will not itself decay to any other state. The vacuum electric field strength *ℰ_C_* —which scales the light–matter interaction strength according to Eq. (A4)—is sampled for a range of values: *ℰ_C_* ∈ [0,6] GV/m. After 500 fs of time evolution, the remaining population in the system (the trace of $\hat{\rho }$) is recorded. This is the portion that has not been removed by absorbing potentials, and it measures the stability of the MgH^+^ molecule over the range of light–matter interaction strengths. The mean lifetime of the field excitation, *τ* = 1/*k*, is then varied over eight orders of magnitude.

**Fig. 3:**
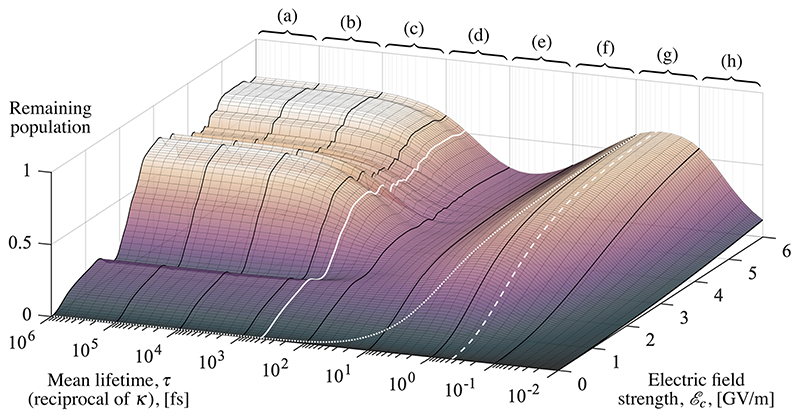
The remaining population on the vertical axis is the trace of $\hat{\rho }$, 500 fs of time evolution after the initial excitation to a dissociative state, and it measures the stability of the MgH^+^ molecule. The electric field strength, *ℰ_c_*, determines the strength of the light–matter coupling in the interaction Hamiltonian (A3). Mean lifetime, *τ*, refers to that of the field excitation from the cavity structure, and *τ* = 1/*k*, where *k* is the decay rate from the Lindblad equation (1). Black thicker lines partition the lifetime parameter into eight sectors [(a)–(h)], each corresponding to one order of magnitude. For reference, the white solid line in sector (d) marks the duration for time evolution (500 fs). The white dashed line in sector (g) marks the time duration for light to cross the length of a Fabry–Perot type cavity (0.5 fs). The white dotted line crossing several sectors marks the points where the Rabi splitting is roughly equal to the full width at half maximum broadening of the field excitation, Ω_*R*_(*q*) ≈ Γ. On the left-hand side of this line resides the strong coupling regime where Ω_*R*_(*q*) ≫ Γ.

Figure 3 shows the population data from a batch of calculations, plotted on a two-dimensional grid. The photon lifetime *τ* is divided into eight sectors, one for each order of magnitude, from 10^5^ fs in sector (a) to 10^–2^ fs in sector (h). For comparison, two relevant times are marked with one white solid line and one white dashed line. The solid line identifies the total duration of time evolution, which corresponds to 500 fs. The dashed line at 0.5 fs identifies the time it takes light to travel across a Fabry–Pérot type cavity with length λ/2 ≈ 140 nm. For such cavities, shorter lifetimes are not physical.

The features calling for explanations in Fig. 3 occur exclusively at higher electric field strengths. For small values, on the other hand, *ℰ_c_* ≲ 1 GV/m, the initial molecular excitation into the dissociative, but non-decaying, state |0, C〉 does not exchange enough population with other dipole-coupled states, and essentially all the population is absorbed during the first 50 fs. This is the expected behavior of a free, dissociating molecule. The following discussion will therefore only consider the behavior of a molecule coupled to the field mode, i.e., *ℰ_c_* ≳ 2 GV/m.

**Fig. 4:**
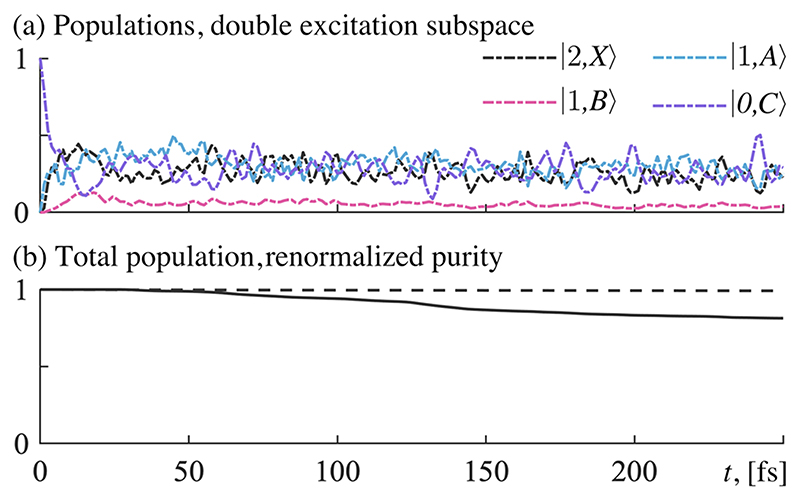
Time evolution data for *ℰ_c_* = 3.0 GV/m and *τ* = 3.6 × 10^4^ fs [sector (b) in Fig. 3]. States not shown have a peak population of less than 0.002. (a) Populated states in the double excitation subspace. (b) Solid line shows the total population, and the dashed line shows the renormalized purity.

In **sectors (a) and (b)** (Fig. 3), where lifetimes are long (*τ* on the order of 10^5^ fs or 10^4^ fs), the molecule is significantly stabilized. This parameter regime can be safely considered as strong coupling. The mean lifetime here is long enough for decay processes to be negligible, and the system behaves as if the lifetime was infinite (or *k* = 0). In these sectors, a sharp rise in molecular stability can be observed as the electric field strengths go beyond 2 GV/m. The stability then plateaus and oscillates around ∼0.7. The cause for the rise in stability can be attributed to the growing Rabi-splitting due to the stronger light–matter coupling.^39,40^ This increases the energy difference between the polaritonic states, which suppresses the transfer of population between them, and stabilizes the molecule. The observed small-scale oscillations are understood as a consequence of interference effects between nuclear wave packets in the crossing regions.^41^ The low rate of decay in these sectors retains the population in the double excitation subspace and the state does not decohere. Time evolution data from sector (b) is shown in Fig. 4.

In **sector (c)**, where the mean lifetime is measured in thousands of femtoseconds (*τ* on the order of 10^3^ fs), the impact of photon decay starts to become noticeable. However, when compared to the infinite lifetime case, the sector is still qualitatively similar to a varying *ℰ_c_*. The error introduced by an infinite lifetime approximation, in sector (c), can be quantified by population deviations from the case of *τ* = ∞. To get the worst-case error in the sector for the population, the lifetime is fixed at its shortest, *τ* = 1000 fs, and the deviation from *τ* = ∞ is calculated for all such points, which gives an average deviation of ~0.07.

The analysis is repeated for a lifetime one order of magnitude larger than the timescale of the studied phenomena. Here, 500 fs of time evolution gives *τ* = 5000 fs. The average deviation is then calculated to ~0.02, which can be an acceptable deviation for many applications.

In **sector (d)**, with mean lifetimes on the order of hundreds of femtoseconds (*τ* on the order of 100 fs), the nuclear dynamics is now on the same timescale as the photon decay and cannot be neglected anymore. A small change in the lifetime now changes the stability of the molecule. In sectors (a)–(c), the long lifetime implies that dissociation is primarily happening in the double excitation subspace but this changes in sector (d). The decay rate is now sufficient for the system to also dissociate from the single excitation and ground-state subspaces via the states |0,*A*〉, |0,*B*〉, |1,*X*〉, and |0,*X*〉. For *ℰ_c_* ≳ 2.5 GV/m, this type of dissociation dominates in sector (d). The loss of coherence in the state $\hat{\rho }$(*t*) results in a suppression of the interference effects and the finer oscillations are dampened. The trapping in polariton states that has contributed to the stabilization now becomes less efficient. The photon decay projects the wave packet partially onto unbound vibrational eigenstates in the single excitation subspace, as well as the ground state. The population remaining in these unbound states for long enough will then contribute to dissociation.

**Fig. 5:**
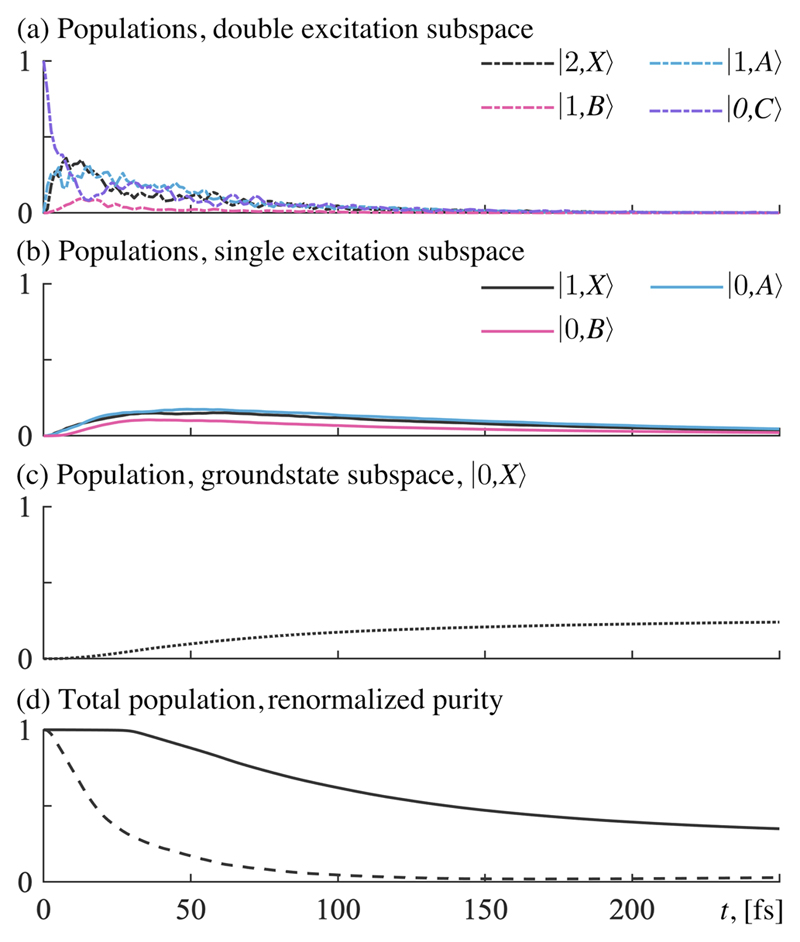
Time evolution data for *ℰ_c_* = 3.0 GV/m and *τ* = 48 f [sector (e) in Fig. 3]. All states are noticeably populated. (a) Populated states in the double excitation subspace. (b) Populated states in the single excitation subspace. (c) Populated states in the ground-state subspace. (d) Solid line shows the total population, and the dashed line shows the renormalized purity.

In **sector (e)**, where the lifetime is on the order of tens of femtoseconds (*τ* on the order of 10^1^ fs), the local minimum in Fig. 3 can be understood as an optimum for dissociation via both the single excitation subspace and the ground state subspace. All subspaces are populated just long enough such that each subspace can contribute to the dissociation. In this sector, the behavior of the system is dominated by effects from decay, and we are now firmly in the dissipative regime (see Fig. 5 for population data from time evolution in the product states).

**fig. 6:**
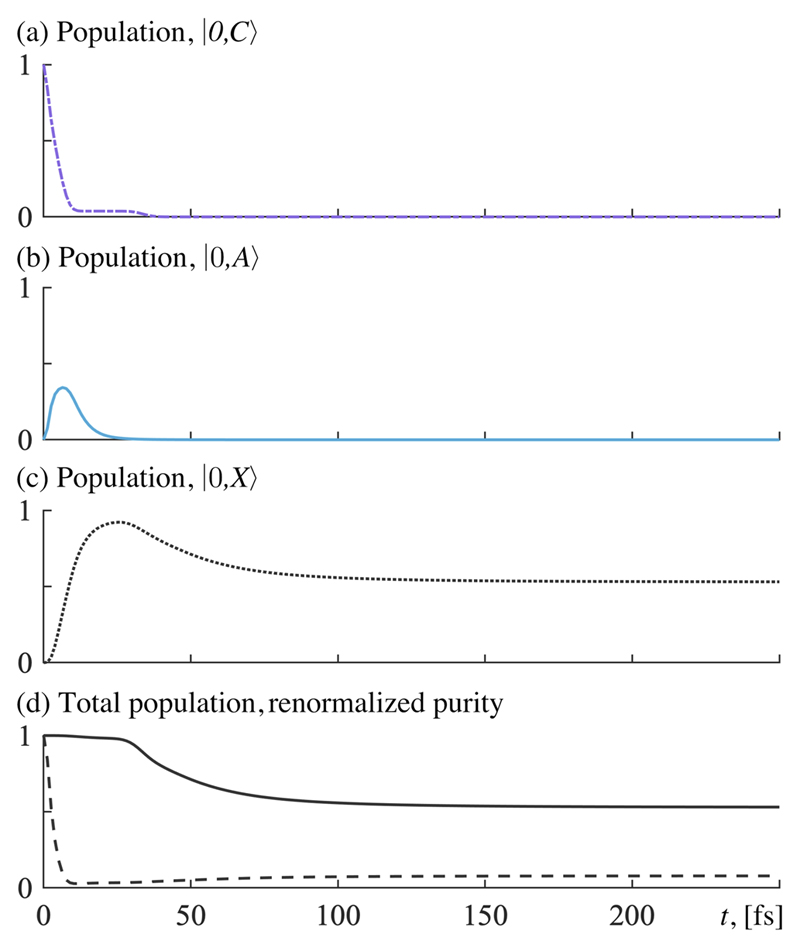
Time evolution data for *ℰ_c_* = 3.0 GV/m and *τ* = 0.58 fs [sector (g) in Fig. 3]. States not shown have a peak population of less than 0.08. (a) Populated states in the double excitation subspace. (b) Populated states in the single excitation subspace. (c) Populated states in the ground-state subspace. (d) Solid line shows the total population, and the dashed line shows the renormalized purity.

In **sectors (f) and (g)**, where the photon lifetime is on the order of femtoseconds or tens of femtoseconds (*τ* on the order of 10^0^ fs or 10^–1^ fs), the lifetime is short enough that the population decays into the ground state before there is enough time to contribute to dissociation from the other subspaces. The time evolution is shown in Fig. 6, where the initial population in |0,*C*⟩ decays almost instantaneously to the mostly bound vibrational states in 0, *X*⟩ (via |0,*A*⟩).

**Fig. 7:**
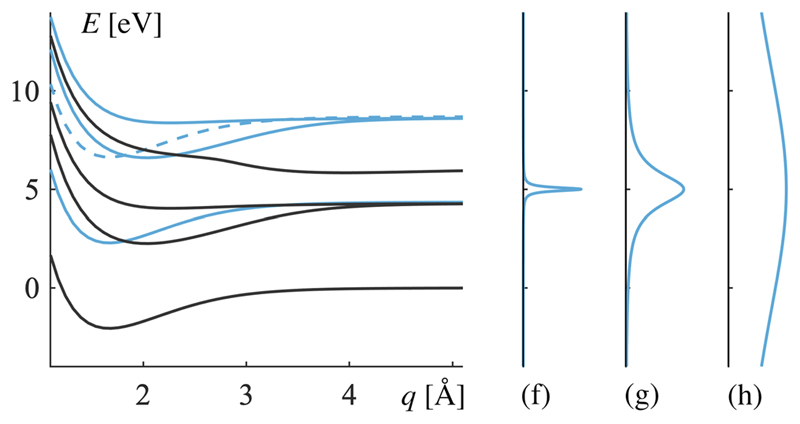
The left-hand side shows the basis states for the system (the same as in Fig. 2). States in blue have some of its energy in the field mode. With the finite photon lifetime, their energy will spread over a superposition of energies in a Lorentzian broadening. The degree of broadening is shown on the right-hand side, plotted on the same energy scale as the left-hand side (and, for clarity, renormalized to equal amplitudes). Graph (f) corresponds to the lifetime of *τ* = 3.2 fs, which is the logarithmic center of sector (f) in Fig. 3, thus estimating the typical energy broadening that sector. The pattern continues, with graph (g) estimating the broadening in sector (g) and graph (h) estimating the broadening in sector (h). The dashed state has two photons and thus twice the broadening of solid blue states.

In **sectors (g) and (h)**, where the lifetime of field excitations is on the order of tens or hundreds of femtoseconds (*τ* on the order of 10^–1^ fs or 10^–2^ fs), a new phenomenon has to be introduced to understand why the molecular stability is declining for the fastest decay. Basis states with a non-zero photon number inherit the relevant lifetime, and here, it is short enough for the energy of these states to become a non-negligible superposition of energies according to a Lorentz distribution with the full width at half maximum Γ = *ħK.* Or put differently, states with short lifetimes experience energy broadening. Which states are affected and the extent of this broadening are shown in Fig. 7. The superposition of energies means that states that were otherwise resonantly dipole-coupled acquire an increasing average detuning, and population transfer is suppressed. The outcome is a smaller spectral overlap and a diminished population transfer between the (sharp) initial state |0, *C*⟩ and the (broadened) decaying states. This keeps most of the population on the potential energy surface of |0, *C*⟩, where it is quickly absorbed at the end of the grid, realizing the declining stability of the MgH^+^ molecule that is most pronounced in sector (h) of Fig. 3.

The increase in energy broadening from Fig. 7 implies that additional states will overlap in energy, and thus, their interactions should be included in the model, which occurs about half-way through sector (g) and certainly in sector (h). This means that the initial assumption about independent subspaces (see Fig. 2) will start to break down. Fortunately, the decline in population seen in sector (h) from Fig. 3 is explained by the weaker coupling between the non-broadening initial state |0, *C*⟩ and the decaying states. This suppressio3n will be unaffected by any missing couplings, and therefore, the qualitative behavior with a decline in stability for very short lifetimes will still hold.

**Fig. 8:**
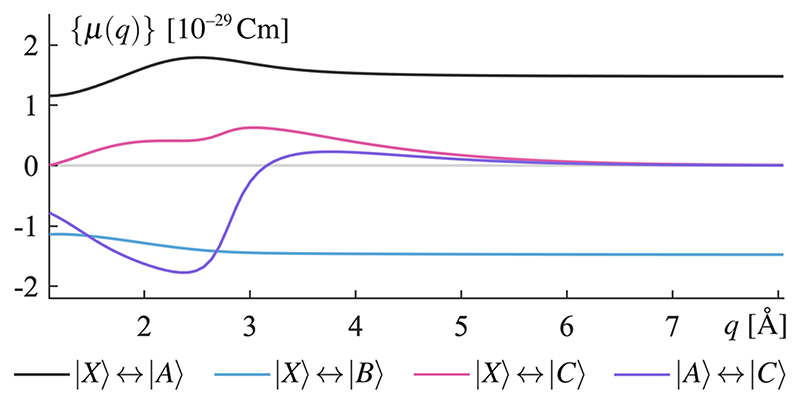
Four transition dipole moments are used in the calculations. |*X*⟩ ↔ |*A*⟩ |*X*⟩ ↔ |*B*⟩, and |*A*⟩ ↔ |*C*⟩ are resonant with the cavity mode frequency and thus included in the interaction Hamiltonian [Eq. (A3)]. The transition |*X*⟩ ↔|*C*⟩ is used to create the initial excited state.

Changes in chemical properties due to the interaction with lossless optical cavities have been under intense theoretical study in the last decade.^1^,15 However, from this study, it is clear that long lifetimes are not a prerequisite for interesting modifications of chemical properties. In Fig. 3, a white dotted line is drawn where the energy width of the field excitation Γ = *ħ_K_* is roughly equal to the Rabi splitting Ω_*R*_(*q*) = 2*ℰ_c_* *μ*(*q*). Transition dipole moments are shown in Fig. 8 as functions of *q*. For finding Ω_*R*_(*q*) ≈ Γ, the transition dipole moment is thus estimated as *μ* =1 × 10^–29^ cm, and a white dotted line for Ω_*R*_ = Γ can be drawn. Some orders of magnitude to the left of this line lie the strong coupling regime, where *Ω_R_*(*q*) ≫ Γ, and to its right, energy broadening is the dominant effect. Still, an equally significant modification of MgH^+^ stability is observed in this regime.

Using the same estimate for the transition dipole moment (*µ* = 1 × 10^–29^ cm), the entire parameter regime shown in Fig. 3 falls just below the ultrastrong coupling regime (where *ℰ*_*c*_ *µ*/*ω*_c_ > 0.1), thus motivating the approximations made in the Hamiltonian (see Appendix A).

## V Conclusion

We have studied the photostability of MgH^+^ in a lossy cavity. The dynamics have been simulated by performing nuclear wave packet dynamics via the Lindblad equation. This approach includes the vibronic decoherence caused indirectly by the loss of photons from the cavity. The studied parameter range includes decay rates inside and outside the strong coupling regime.

Deep in the strong coupling regime, for cavity lifetimes longer than the nuclear dynamics (*τ* ≫ 100 fs), stabilization of MgH^+^ is achieved through the formation of well-separated polariton states. Interference effects at the curve crossing can be observed due to the nearly fully coherent time evolution. For cavity decay rates on the order of tens of femtoseconds, the stabilization effect decreases and the aforementioned interference effects disappear. Even though this regime can still be regarded as the strong coupling regime, the coherent wave packet time evolution now competes with the effects from dissipation. The separation of polaritonic states, which is responsible for the stabilization, is now affected by the photon decay.

Shortening the photon lifetime even further, down below 1 fs, increases the stabilization again, as the molecule is rapidly funneled back into its ground state of the system. Our results suggest that, for an optimal photon lifetime in this region, low Q-factor cavities may facilitate the control of photochemical reactions,21 where severalcompeting mechanisms are responsible for the observed phenomena: The population is transferred efficiently between the polariton states, which may be interpreted as an optimized spectral overlap of the cavity mode^42^ with the energy width of the nuclear wave packet. The short photon lifetime contributes to the cooling of the system. With fast enough decay, the molecule can dissipate the energy stored in the electronic excitation before dissociation takes place. The combination of both effects results in an optimal stabilization of MgH^+^ in this particular configuration.

It is worth noting that the stability optimum is at the border of the strong coupling regime, which has also been found in other studies.^21^ This suggests that it may be an interplay of strong coupling and cavity cooling, which is required to explain polaritonic chemistry experiments.

## Supplementary Material

Appendix

## Data Availability

The data that support the findings of this study are available from the corresponding author upon reasonable request.
