# Supplementary material for: Simulating photodissociation reactions in bad cavities with the Lindblad equation
Source: J Chem Phys. Author manuscript; Available in PMC 2021 Feb 11. (PMC7116731; doi:10.1063/5.0033773)
Supplement: Appendix [file EMS115640-supplement-Appendix.pdf]

APPENDIX A: THE HAMILTONIAN

The molecular Hamiltonian is here expressed in terms of the four lowest electronic states $\{|X\rangle, |A\rangle, |B\rangle, |C\rangle\}$,

$$\hat{H}_m = -\frac{\hbar^2}{2M}\frac{\mathrm{d}^2}{\mathrm{d}q^2} + V_X(q)|X\rangle\langle X| + V_A(q)|A\rangle\langle A| + V_B(q)|B\rangle\langle B| + V_C(q)|C\rangle\langle C|. \tag{A1}$$

The reduced mass, M, of the MgH^+ molecule is 1763.2 m_e. 1D potential energy surfaces $\{V_X(q), \ldots, V_C(q)\}$ are calculated under the Born–Oppenheimer approximation with the program package Molpro[33] at the CASSCF(12/10)/MRCI/ROOS level of theory[34,35] and interpolated using splines to a grid with 96 grid points. Wave packets approaching the right-hand side of the grid are removed by Gaussian-shaped absorbing potentials. How much norm that has been removed is stored as a function of time, separately for each product state (see Fig. 1).

The frequency of the fundamental cavity mode ω_c is chosen to match a bright transition of the Mg atom (at 285 nm).[9] The cavity mode is modeled in the Fock basis $\{|0\rangle, |1\rangle, |2\rangle, \ldots\}$. With the photonic creation and annihilation operators, $\hat{a}^\dagger$ and $\hat{a}$, respectively, the cavity Hamiltonian is formed,

$$\hat{H}_c = \hbar\omega_c\,\hat{a}^\dagger\hat{a}. \tag{A2}$$

Light–matter interactions are modeled under the dipole and rotating wave approximations. Molecular transitions resonant with the cavity mode frequency are $|X\rangle \leftrightarrow |A\rangle$, $|X\rangle \leftrightarrow |B\rangle$, and $|A\rangle \leftrightarrow |C\rangle$ (see Fig. 1 for their potential energy surfaces),

$$\hat{H}_{cm} = \mathscr{E}_c\mu_{XA}(q)\Big(\hat{a}^\dagger|X\rangle\langle A| + \hat{a}|A\rangle\langle X|\Big) + \mathscr{E}_c\mu_{XB}(q)\Big(\hat{a}^\dagger|X\rangle\langle B| + \hat{a}|B\rangle\langle X|\Big) + \mathscr{E}_c\mu_{AC}(q)\Big(\hat{a}^\dagger|A\rangle\langle C| + \hat{a}|C\rangle\langle A|\Big). \tag{A3}$$

The transitions $|A\rangle \leftrightarrow |B\rangle$, $|B\rangle \leftrightarrow |C\rangle$, and $|X\rangle \leftrightarrow |C\rangle$ are not resonant with the cavity mode frequency, but the transition $|X\rangle \leftrightarrow |C\rangle$ is used for creating the initial excited state. The transition dipole moments $\{\mu(q)\}$ are obtained at the same level of theory as the potential energy surfaces, and Fig. 8 shows the ones that are used in the model. For a minimal model of the molecule, permanent dipole moments and self-energy are omitted. The vacuum electric field strength, $\mathscr{E}_c$, determines the strength of the light–matter interaction and depends on the mode volume of the cavity structure, V,

$$\mathscr{E}_c = \sqrt{\frac{\hbar\omega_c}{2\epsilon_0 V}}. \tag{A4}$$

APPENDIX B: SUMMING LINDBLAD OPERATORS AHEAD OF TIME EVOLUTION

To do time evolution with the Lindblad equation (1), all $\{\hat{L}_n\}$ operators must be stored in memory, and given N such operators, $6N$ matrix multiplications are required for each time step. A method for reducing memory requirements and computational cost is demonstrated here. Memory storage is reduced from N matrices to 2. The computational cost goes from $6N$ matrix multiplications to four matrix multiplications and two operations where off-diagonal elements are set to zero. Three assumptions are used, which are fulfilled in this study.

Assumption 1. In the basis employed for the computation, each Lindblad operator is decaying a single basis state to another basis state. □

Assumption 2. There are no Lindblad pure de-phasing operators in the model. □

Assumption 3. Lindblad operators and decay rates are time-independent. □

If assumptions 1 and 2 do not hold, this method may still be useful in combination with a change of basis and/or treating de-phasing operators separately.

There are two useful notations for the set of all Lindblad operators. The most minimal notation is to simply enumerate them,

$$\Big\{\hat{L}_n : n \in [1, \ldots, N]\Big\}. \tag{B1}$$

However, according to assumption 1, each operator involves only two states from the employed basis $\{|e_i\rangle\}$. Thus, Lindblad operators can also be expressed in terms of the basis states that comprise them,

$$\Big\{\hat{L}_{ij} : \hat{L}_{ij} := |e_i\rangle\langle e_j|\Big\}. \tag{B2}$$

The associated decay rates are denoted as $\{\kappa_n\}$ or $\{\kappa_{ij}\}$, respectively. Depending on whether the emphasis is on the number of operators or on the basis states that comprise them, these two notations will be used interchangeably in the following.

The focus is on treating the Lindblad operators inside the sum of the Lindblad equation,

$$\partial_t\hat{\rho} = -\frac{\mathrm{i}}{\hbar}\Big[\hat{H}, \hat{\rho}\Big] + \sum_n \kappa_n\Big(\hat{L}_n\hat{\rho}\hat{L}_n^\dagger - \frac{1}{2}\Big[\hat{L}_n^\dagger\hat{L}_n, \hat{\rho}\Big]_+\Big). \tag{B3}$$

These two terms in Eq. (B3) are performing the functions of re-populating states, and depopulating states, in that order of appearance.

© Author(s) 2020

The first term, responsible for re-populating states, can unfortunately not be factorized,

$$\sum_n \kappa_n \hat{L}_n \hat{\rho} \hat{L}_n^\dagger \neq \left(\sum_n \sqrt{\kappa_n}\hat{L}_n\right)\hat{\rho}\left(\sum_n \sqrt{\kappa_n}\hat{L}_n\right)^\dagger. \tag{B4}$$

However, we treat it as if it could be anyway and define the left parenthesis from Eq. (B4) as $\hat{S}_1$,

$$\hat{S}_1 := \sum_n \sqrt{\kappa_n}\,\hat{L}_n \equiv \sum_{\{ij\}} \sqrt{\kappa_{ij}}\,\hat{L}_n. \tag{B5}$$

The multiplication from Eq. (B4) is then expanded,

$$\begin{aligned}\hat{S}_1\hat{\rho}\hat{S}_1^\dagger = &\left(\kappa_1\hat{L}_1\hat{\rho}\hat{L}_1^\dagger + \sqrt{\kappa_1\kappa_2}\,\hat{L}_1\hat{\rho}\hat{L}_2^\dagger + \sqrt{\kappa_1\kappa_3}\hat{L}_1\hat{\rho}\hat{L}_3^\dagger + \cdots\right)\\ &+\left(\sqrt{\kappa_2\kappa_1}\,\hat{L}_2\hat{\rho}\hat{L}_1^\dagger + \kappa_2\hat{L}_2\hat{\rho}\hat{L}_2^\dagger + \sqrt{\kappa_2\kappa_3}\,\hat{L}_2\hat{\rho}\hat{L}_3^\dagger + \cdots\right)\\ &+\left(\sqrt{\kappa_3\kappa_1}\,\hat{L}_3\hat{\rho}\hat{L}_1^\dagger + \sqrt{\kappa_3\kappa_2}\,\hat{L}_3\hat{\rho}\hat{L}_2^\dagger + \kappa_3\hat{L}_3\hat{\rho}\hat{L}_3^\dagger + \cdots\right) + \cdots. \end{aligned}\tag{B6}$$

Terms with a single κ_n are identical to the desired terms from the Lindblad equation (B3), but there are also several unwanted cross-terms. Expressing $\hat{\rho}$ in the employed basis $\{|e_i\rangle\}$ and using the result that all operators $\{\hat{L}_n\} \equiv \{\hat{L}_{ij}\}$ are of the form of Eq. (B2), we simplify the operator for each type of term, beginning with the single κ_n term,

$$\hat{L}_{ij}\hat{\rho}\hat{L}_{ij}^\dagger = |e_i\rangle\langle e_j|\left(\sum_{k,l}|e_k\rangle\langle e_l|\rho_{kl}\right)|e_j\rangle\langle e_i| = |e_i\rangle\langle e_i|\rho_{jj}. \tag{B7}$$

These desired terms thus correspond to increasing population in diagonal matrix elements of $\hat{\rho}$ (due to $|e_i\rangle\langle e_i|$), in proportion to other diagonal matrix elements of $\hat{\rho}$ (due to ρ_{jj}). Then, the cross-terms are simplified as

$$\hat{L}_{ij}\hat{\rho}\hat{L}_{mn}^\dagger = |e_i\rangle\langle e_j|\left(\sum_{k,l}|e_k\rangle\langle e_l|\rho_{kl}\right)|e_n\rangle\langle e_m| = |e_i\rangle\langle e_m|\rho_{jn}. \tag{B8}$$

The $\{\hat{L}_{ij}\}$ operators are required to be orthogonal, which means that either $i \neq m$ or $j \neq n$. That is, either $|e_i\rangle\langle e_m|$ is an off-diagonal matrix element after matrix multiplication or ρ_{jn} is an off-diagonal matrix element before matrix multiplication (or indeed both).

Setting off-diagonal elements of $\hat{\rho}$ to zero both before and after matrix multiplication with $\hat{S}_1$ will therefore remove only the cross-terms from Eq. (B6). Let $\mathfrak{D}[\cdot]$ be this operation, which sets off-diagonal matrix elements to zero in the employed basis. The initial sum over multiple matrix multiplications can now be rewritten using $\hat{S}_1$,

$$\sum_n \kappa_n\hat{L}_n\hat{\rho}\hat{L}_n^\dagger = \mathfrak{D}\left[\hat{S}_1\hat{\mathfrak{D}}[\hat{\rho}]\hat{S}_1^\dagger\right]. \tag{B9}$$

In contrast to the re-populating term from the Lindblad equation (B3), the other term, responsible for depopulation, can be factorized,

$$\sum_n -\kappa_n\frac{1}{2}\left[\hat{L}_n^\dagger\hat{L}_n,\hat{\rho}\right]_+ = \left(-\frac{1}{2}\sum_n \kappa_n\hat{L}_n^\dagger\hat{L}_n\right)\hat{\rho} + \hat{\rho}\left(-\frac{1}{2}\sum_n \kappa_n\hat{L}_n^\dagger\hat{L}_n\right). \tag{B10}$$

Defining the left parenthesis above as the operator $\hat{S}_2$, we can reduce this to two matrix multiplications,

$$\hat{S}_2 := -\frac{1}{2}\sum_n \kappa_n\hat{L}_n^\dagger\hat{L}_n \equiv -\frac{1}{2}\sum_{\{ij\}}\kappa_{ij}\hat{L}_{ij}^\dagger\hat{L}_{ij}. \tag{B11}$$

With the definition of $\hat{S}_1$ and $\hat{S}_2$ from Eqs. (B5) and (B11), they are calculated once ahead of time evolution and stored in memory, and the set $\{\hat{L}_n\}$ can be discarded. Together with the operation $\mathfrak{D}[\cdot]$, the initial sum from Eq. (B3) with $6N$ matrix multiplications is reduced to four matrix multiplications and two $\mathfrak{D}$-operations where off-diagonal elements are set to zero,

$$\sum_n \kappa_n\left(\hat{L}_n\hat{\rho}\hat{L}_n^\dagger - \frac{1}{2}\left[\hat{L}_n^\dagger\hat{L}_n,\hat{\rho}\right]_+\right) = \mathfrak{D}\left[\hat{S}_1\hat{\mathfrak{D}}[\hat{\rho}]\hat{S}_1^\dagger\right] + \hat{S}_2\hat{\rho} + \hat{\rho}\hat{S}_2^\dagger. \tag{B12}$$

APPENDIX C: NUMERICAL METHOD AND VERIFICATION

The Lindblad equation is solved numerically, using the ode45[36] (Runge–Kutta) differential equation solver, as implemented in Octave,[37] hereafter referred to as the Octave method. Verification and accuracy benchmarks of the Octave method are done by comparison to time evolution with the Schrödinger equation, in the in-house QDng package, using the Chebyshev propagator,[38] hereafter referred to as the QDng method. This QDng method uses 512 grid points and a fixed duration time step set at 0.0242 fs, previously determined to have high accuracy in similar systems when compared to even shorter time steps.[9] The Octave method uses 96 grid points and a variable duration time step. Accuracy is here specified with a 1×10^{-6} absolute tolerance and 1×10^{-3} relative tolerance. For both methods, we calculate the remaining population after 500 fs of time evolution (i.e., the same data that make up our main result in Fig. 3). The QDng method requires there to be no photon decay; thus, $\kappa = 0$ in the Octave method. A comparison of the two methods is shown in Fig. 9.

At a standard deviation in the remaining population of 0.0086, the agreement between the two methods is satisfactory. Noticeable deviations occur for higher field strengths, where the impact of interference effects causes some disagreement. However, the overall profile of the oscillations is still accurately tracked.

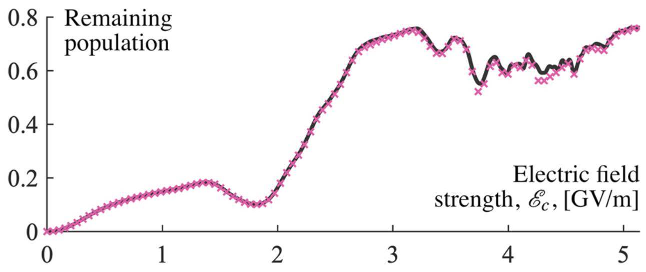

FIG. 9. Data for method accuracy benchmark. The remaining population in the system after 500 fs of time evolution. The black curve is obtained from 1000 QDng, Chebyshev propagator, calculations (QDng method) and considered the baseline for accuracy comparisons. Pink crosses are the results from 100 Octave, ode45, calculations (Octave method).

© Author(s) 2020

APPENDIX D: TIME-RESOLVED DATA FROM SIMULATIONS

Details from time evolution, for three data points in the parameter range, are shown in Figs. 4, 5, and 6. The first data point is in the polaritonic strong coupling regime, with parameters $\mathscr{E}_c$ = 3.0 GV/m and $\tau = 3.6 \times 10^4$ fs, directly after the sudden rise in sector (b) of Fig. 3. The second data point has parameters $\mathscr{E}_c$ = 3.0 GV/m and τ = 48 fs, which puts it in the low stability region of sector (e). The third data point has parameters $\mathscr{E}_c$ = 3.0 GV/m and τ = 0.58 fs, which is a data point on top of the high stability region in sector (g) of Fig. 3. At each data point, populations are plotted individually for each product state (see Fig. 2 for the states). States that are not noticeably populated (peak population less than 0.08) are not shown. The total population remaining in the system is also shown, along with a renormalized purity. The renormalization is done to eliminate loss of purity caused directly from the loss of norm. The full time evolution is 500 fs, but plots are limited to the first 250 fs where the influential processes are taking place.

© Author(s) 2020
